# Immunosuppression and herpes viral reactivation in intensive care unit patients: one size does not fit all

**DOI:** 10.1186/s13054-017-1803-1

**Published:** 2017-08-26

**Authors:** Julien Textoris, François Mallet

**Affiliations:** 1 EA7426 “Pathophysiology of Injury-Induced immunosuppression”, Hospices Civils de Lyon—Université Claude Bernard Lyon 1—bioMérieux, Lyon, France; 2 Anesthesiology and Critical Care Medicine, Hospices Civils de Lyon—Université Claude Bernard Lyon 1, Lyon, France

**Keywords:** Viral reactivation, Herpes, Immunosuppression

More than 20 years after the initial description of cytomegalovirus (CMV) pneumonia in “non-immunocompromised” ICU patients by Papazian et al. [1], the treatment of herpes viruse reactivation in ICU is still a matter of debate. Recently, Mirouse et al. [2] reported a unique cohort of varicella-zoster virus (VZV)-related community acquired pneumonia in the ICU over 20 years. This highlighted that VZV infections in ICU patients are rare, with roughly a hundred cases over 20 years, in 29 French ICUs. Conversely, addressing a wider range of herpes viruses, two major papers recently reported a high frequency of viral reactivation in critically ill patients, and both showed variable plasma levels of various herpes viruses (Table 1) [3, 4]. Interestingly, almost 30% of the patients had multiple viremia events, and this viremia usually lasted until ICU discharge. The timing of viral reactivation was also informative, with herpes simplex virus (HSV)1 and Epstein-Barr virus (EBV) being detected earlier in the ICU course than CMV or human herpesvirus (HHV)6.

**Table 1:** Cumulative percentages of reactivation of various herpes viruses in septic patients during ICU stay

Virus	Walton et al. [3]	Ong et al. [4]
VZV	-	0.6%
HSV1	14%^a^	26% (31%^a^)
HSV2	-	4%
HHV6	10%	24%
CMV	24%^a^	18% (27%^a^)
EBV	53%	48%

^a^Percentage in HSV1 or CMV seropositive patients only

Several herpes viruses have been associated repeatedly with mortality and the occurrence of secondary infections. The most validated association is between CMV and mortality, either in ICU or at various times post-ICU [3–5]. In the MARS cohort, this association remained significant after adjusting for confounders, time-dependent bias, and competing risks [4]. Importantly, this association between CMV viremia and ICU mortality was significant (adjusted sdHR = 3.2 [1.4–7.1]) while taking into account other viral reactivation, which was never considered before [4]. Multiple reactivations might also suggest increased severity since the association of HHV6 and CMV reactivation was also associated with an increased risk of death in critically ill patients [6]. Association between EBV reactivation and mortality was also described [4, 7]. Data are less clear for other neurotropic herpes viruses such as HSV2 or VZV, which exhibit lower reactivation rates. Finally, in septic patients, CMV/EBV and HSV1 have also been associated with an increased rate of secondary fungal and bacterial infections, respectively [4]. This last result supports the hypothesis that immunosuppression might play an important role in this reactivation.

Most data related to critically ill patients made a clear distinction between immunocompromised (mainly oncologic treated patients, solid or bone marrow transplants) and so-called *immunocompetent* patients. However, we know that 30 to 50% of these critically ill patients exhibit signs of immunosuppression [8]. Clearly, the wording *immunocompetent*—to identify a category of patients that do not exhibit a severe iatrogenic or congenital immunosuppression—is misleading and it is time to propose several levels of immunosuppression (in terms of depth or type of immunosuppression) to better reflect the various levels of depressed immune status in ICU patients. Herpes viremia might therefore reflect either a lack of a latent virus control or a true clinical viral infection.

The ability to describe several types or levels of immunosuppression is important and supports a wider use of quantitative tools to measure viral titers in addition to host response biomarkers of the immune status. Considering the fact that many bone marrow transplant patients reactivate VZV [9] whereas VZV reactivation is almost absent in critically ill patients (this is supported also by the paucity of VZV severe pneumonia cases reported by Mirouse et al. over 20 years [2]), monitoring a panel of herpes viruses might also provide information about the type/depth of immunosuppression, and should be explored in more details. More generally, a quantitative and qualitative altered state of the plasma virome was observed in tacrolimus [10] or HIV-1 [11] induced immunosuppression, notably highlighting the correlation of the anellovirus viral load with the extent of immunosuppression.

Quantitative tools should allow us to define several thresholds to discriminate between (1) non-significant viral load, (2) viral “reactivation” as a marker of immunosuppression, and (3) high viral loads supporting a true viral infection requiring treatment. Such stratification with various levels of viremia might also be key to stratify patients for trials assessing the effectiveness of prophylactic/pre-emptive/or curative antiviral treatment in ICU patients [12]. It might also be part of the stratification tools/criteria used to select appropriate immunotherapy to critically ill patients [13, 14]. It is important to consider that the global virome (including endogenous retroviruses), as part of the collective microbiome (and in addition to intrinsic and environmental factors), shape and regulate our immune system [15]. As commensal viruses might benefit the host [16], we should carefully consider the administration of antivirals, which will profoundly manipulate our virome and may end up doing more harm than good. Knowledge and tools are needed to better assess the host response, guide therapeutics, and avoid disruption in virome/microbiome–host homeostasis.
